# Enhancing Osteoporosis Treatment through Targeted Nanoparticle Delivery of Risedronate: In Vivo Evaluation and Bioavailability Enhancement

**DOI:** 10.3390/pharmaceutics15092339

**Published:** 2023-09-18

**Authors:** Zoya Saifi, Sadat Shafi, Tanya Ralli, Shreshta Jain, Divya Vohora, Showkat Rasool Mir, Abdulsalam Alhalmi, Omar M. Noman, Ahmad Alahdab, Saima Amin

**Affiliations:** 1 Department of Pharmaceutics, School of Pharmaceutical Education and Research, Jamia Hamdard, New Delhi 110062, India; zoya783@gmail.com (Z.S.); tanyaralli94@gmail.com (T.R.); asalamahmed5@gmail.com (A.A.); 2 Department of Pharmacology, School of Pharmaceutical Education and Research, Jamia Hamdard, New Delhi 110062, India; sadat_shafi@yahoo.com (S.S.); shreshtha.jn@gmail.com (S.J.); dvohora@jamiahamdard.ac.in (D.V.); 3 Department of Pharmacognosy and Phytochemistry, School of Pharmaceutical Education and Research, Jamia Hamdard, New Delhi 110062, India; srmir@jamiahamdard.ac.in; 4 Department of Pharmacognosy, College of Pharmacy, King Saud University, P.O. Box 2457, Riyadh 11451, Saudi Arabia; onoman@ksu.edu.sa; 5 Institute of Pharmacy, Clinical Pharmacy, University of Greifswald, Friedrich-Ludwig-Jahn-Str. 17, 17489 Greifswald, Germany; ahmad.alahdab@uni-greifswald.de

**Keywords:** osteoporosis, thiolated chitosan, risedronate, hydroxyapatite, nanoparticles

## Abstract

Risedronate-loaded mPEG-coated hydroxyapatite, thiolated chitosan-based (coated) and non-coated nanoparticles were tested for their potential effects in the treatment of osteoporosis. The prepared nanoparticles were evaluated for their bone-targeting potential by inducing osteoporosis in female Wistar rats via oral administration of Dexona (dexamethasone sodium phosphate). In vivo pharmacokinetic and pharmacodynamic studies were performed on osteoporotic rat models treated with different formulations. The osteoporotic model treated with the prepared nanoparticles indicated a significant effect on bone. The relative bioavailability was enhanced for RIS-HA-TCS-mPEG nanoparticles given orally compared to RIS-HA-TCS, marketed, and API suspension. Biochemical investigations also showed a significant change in biomarker levels, ultimately leading to bone formation/resorption. Micro-CT analysis of bone samples also demonstrated that the RIS-HA-TCS-mPEG-treated group showed the best results compared to other treatment groups. Moreover, the histology of bone treated with RIS-HA-TCS-mPEG showed a marked restoration of the architecture of trabecular bone along with a well-connected bone matrix and narrow inter-trabecular spaces compared to the toxic group. A stability analysis was also carried out according to ICH guidelines (Q1AR2), and it was found that RIS-HA-TCS-mPEG was more stable than RIS-HA-TCS at 25 °C. Thus, the results of present study indicated that mPEG-RIS-HA-TCS has excellent potential for sustained delivery of RIS for the treatment and prevention of osteoporosis, and for minimizing the adverse effects of RIS typically induced via oral administration.

## 1. Introduction

Osteoporosis is a pathological condition characterized by a gradual decline in bone mineral density, deterioration of the microstructural integrity, increased susceptibility to fractures, and subsequent reduction in the overall well-being of affected individuals [1]. Bisphosphonates help to prevent further bone deterioration of advanced osteoporosis and thus have been used in the treatment of osteoporosis [2]. Alendronate and risedronate (RIS ) are widely recognized as the primary therapeutic interventions for the prophylaxis and treatment of osteoporosis in postmenopausal females. One of the primary challenges impeding the oral administration of RIS is its limited permeability and inadequate and variable bioavailability, which remains below 1% [3]. The limited bioavailability of RIS necessitates the administration of elevated doses, potentially resulting in the manifestation of adverse effects such as osteonecrosis of the jaws, fever, vein irritation, general aches and pains, and kidney dysfunction [3]; hence, augmenting the bioavailability of RIS is imperative. Consequently, ongoing investigations in drug delivery have prioritized the development of a delivery modality capable of augmenting bone characteristics while utilizing a reduced dose of RIS.

Hydroxyapatite (HA) is abundant in the bone matrix and teeth. Its structural and functional resemblance to natural HA has prompted numerous investigations into using synthetic HA as a bone substitute in various biomedical contexts [4]. HA exhibits a favorable capacity for bone remodeling, characterized by its positive osteogenic activity instead of negatively influencing osteoclast activity [5]. An appreciable enhancement has been observed in the efficacy of HA upon its conversion into particles within the nano-scale range. In a recent investigation conducted by Sun et al., the impact of HA particle size on in vivo osteoblastic cells was examined. The study revealed that therapeutic efficacy was enhanced when HA particles of size within 0.5–3.0 μm were employed [6]. The properties mentioned above hold significant importance due to the perpetual remodeling of bone tissue, wherein bone cells simultaneously replace and remove bone tissue [7].

Chitosan, a polymer of significant interest in biomedical and pharmaceutical sciences, is renowned for its remarkable versatility, but has pH-dependent solubility [8]. Several derivatives were synthesized to address the solubility limitation and enhance chitosan’s mucoadhesive features. These derivatives include trimethylated chitosan [9], mono-N-carboxymethyl chitosan [10], N-sulfochitosan [11], and chitosan-EDTA conjugates [12]. A further modification is based on immobilizing thiol-bearing moieties on the polymeric backbone of chitosan [13]. Thiomer, a hydrophilic macromolecule, possesses free thiol groups within its structural composition. In the field of pharmaceutical science, the thiolation of this particular natural polymer has been found to yield numerous advantages. These benefits encompass various effects, including providing mucoadhesive properties, enhancing mucoadhesive properties, and protein permeation. Thiolated chitosan (TCS) has recently garnered significant attention as a promising pharmaceutical polymer [14].

Based on the above facts, osteotropic drug delivery systems (ODDSs) were proposed as a potential method to enhance the drug’s affinity towards bone tissues [15,16]. In a study by Sahana et al., the therapeutic superiority of novel RIS-HA-loaded nanoparticles (NPs) compared to RIS monotherapy was demonstrated in the context of treating osteoporosis. Specifically, the researchers utilized a rat model of postmenopausal osteoporosis to investigate the efficacy of these treatment modalities [17,18]. In this study, we thoroughly evaluated the effectiveness of RIS-HA-TCS-mPEG nanoparticles developed for the treatment of osteoporosis. Our evaluation encompassed biochemical, pharmacodynamic, and pharmacokinetic analyses to compare the performance of these nanoparticles with that of RIS-HA-TCS, API (active pharmaceutical drug) suspension, and a marketed available drug.

## 2. Materials and Methods

### 2.1. Materials

A sample of RIS sodium, with an average molecular weight of 350.13 g/mol, was obtained as a gift from Jubilant Life Sciences, located in Noida, Uttar Pradesh, India. The mPEG 2000-DSPE (PE 18:0/18:0-PEG, 2000) was obtained from Lipoid, Ludwigshafen, Germany. Hydroxyapatite (HA), possessing a molecular weight of 502.31 g/mol, and thioglycolic acid (TGA), with a molecular weight of 92.12 g/mol, were procured from S.G Enterprises, located in New Delhi, India. Dialysis bags possessing a specific molecular weight cut-off (MWCO) of 12000Da, chitosan with a molecular weight of 750,000Da and 75–85% degree of deacetylation (DDA), sodium tripolyphosphates (TPP), and Dimethylformamide (DMF) with a molecular weight of 73.09 g/mol, were procured from Sigma Aldrich, New Delhi, India. N-hydroxy succinimide (NHS), 1-ethyl-3-(3-dimethylaminopropyl) Carbodiimide Hydrochloride (EDAC), and Ellman Reagents were obtained from Spectro-chem, Mumbai, India. Methanol and acetonitrile of high-performance liquid chromatography (HPLC) grade were obtained from S.D. Fine Chemicals Ltd., Mumbai, India. All additional reagents and chemicals employed in the investigation were of analytical grade.

### 2.2. Preparation of RIS-HA-TCS-mPEG Nanoparticles

The synthesis of RIS-HA-TCS-mPEG nanoparticles was successfully achieved via a sequential four-step reaction process. The synthesis of thioglycolic acid-modified chitosan (TCS) involved a reaction between chitosan and thioglycolic acid. In the subsequent stage, the production of RIS incorporating HA (Hydroxyapatite) was accomplished using the physical adsorption technique. In the third phase of the experiment, the production of nanoparticles was carried out using the ionic gelation technique. This involved the addition of the synthesized RIS-HA conjugates and TCS to facilitate the formation of the nanoparticles. The optimization process of RIS-HA-TCS nanoparticles was carried out using the Box–Behnken design methodology. The optimized RIS-HA-TCS formulation was subjected to PEGylation using mPEG to improve its stability and bioavailability [19]. The identification of the product at each stage was achieved through Fourier Transform Infrared Spectroscopy (FT-IR) and Differential Scanning Calorimetry (DSC) methodologies.

### 2.3. Confocal Laser Scanning Microscope (CLSM)

The experimental animals (female Wistar rats having a weight of 225–250 g) were subjected to an overnight fasting period before the experiment. They were then anesthetized using diethyl ether, and subsequently euthanized via cervical dislocation. The surgical procedure involved the extraction of the small intestine, followed by subsequent slicing and rinsing of a 5 cm segment using the standard saline solution to eliminate any remaining food residues [20]. The intestinal section was carefully ligated at one end to secure closure. Subsequently, the samples, namely a solution of 0.03% Rhodamine B (Rh-B) in water, Rh-B-loaded RIS-HA-TCS formulation, and RIS-TCS-HA-mPEG formulation, were introduced into the intestinal section using a 5 mL syringe. Rhodamine B (Rh-B)-loaded RIS-HA-TCS, and RIS-TCS-HA-mPEG formulations were synthesized using the identical methodology employed for the development of RIS-HA-TCS and RIS-HA-TCS-mPEG formulations, with the exception that Rh-B was introduced during the preparation of the formulation at the last step with gentle homogenization. Subsequently, the sac was fully submerged within a beaker comprising 100 mL of Kreb’s solution. The solution underwent aeration for 4 h, and was subjected to stirring at a rate of 50 revolutions per minute (rpm) at a controlled temperature of 37 ± 1 °C. Following a 4 h incubation period, the loops were longitudinally sectioned, and any surplus dye was eliminated through thorough washing with Kreb’s solution. The intestinal tissues that underwent treatment were affixed onto the glass slides [21]. Rh-B dye penetration depth analysis along the ɀ-axis was conducted using CLSM equipped with an Olympus Fluoview TM FV 1000 system. The fluorescence excitation was achieved using a laser operating at a wavelength of 514 nm [22].

### 2.4. In Vivo Pharmacodynamic Studies

#### 2.4.1. Animals

An in vivo investigation was duly authorized by the Institutional Animal Ethics Committee of Jamia Hamdard, located in New Delhi. The study adhered strictly to the guidelines set forth by the aforementioned committee for the entire duration of the research (Approval no. 1821/CPCSEA). The animal model utilized in this study consisted of female Wistar rats with an average weight ranging from 225 to 250 g. The animals were exposed to standard laboratory settings and supplied with a normal pellet-based diet, i.e., Lipton and unimpeded water availability.

#### 2.4.2. Study Design

To facilitate the pharmacodynamics investigations, eighteen animals were selected for the study. These animals were then divided into six separate groups, each labelled as A, B, C, D, E and F. Before the commencement of the study, the animals were individually weighed; the average weight was 200 ± 20 g. The initial phase involved the induction of osteoporosis in groups B, C, D, E, and F using Dexona (dexamethasone sodium phosphate) at a dosage of 2 mg/kg body weight (Zydus Alidac, Bangalore, India, 2 mg/mL) via subcutaneous injection once weekly for a duration of 4 weeks [23]. The subjects were allowed to remain undisturbed for six weeks, during which osteoporosis development was observed [24]. Group A was designated as the control group, while Group B was designated as the pathogenic control group. The administration of the dosage occurred every week, lasting four consecutive weeks for all the groups, except the control group. The details are given in Table 1.

#### 2.4.3. Biochemical Analysis

After four weeks of induction, the rats underwent diethyl ether anesthesia. Following the procedure, blood samples were obtained from the retro-orbital plexus and precisely collected into desiccated test tubes. The sample underwent centrifugation at a speed of 5000 revolutions per minute (rpm) for 10 min to facilitate the segregation of the serum constituent. The acquired serum was utilized to carry out biochemical analyses. The serum alkaline phosphatase and serum calcium levels were assessed using a commercially accessible standard biochemical kit from Span Diagnostic Ltd. (Surat, India).

#### 2.4.4. Histology of Bone’s Internal Structure

Rats were subjected to sacrifice, following which femur bone was separated from the body. The bones were carefully removed through dissection techniques, and subsequently immersed in a solution of 10% formalin. This was conducted in preparation for the evaluation of the femur’s histological characteristics. The bone specimens underwent a thorough washing process lasting 12 h. This process involved immersing the specimens in three consecutive series of solutions. The first series consisted of 0.01 M phosphate-buffered saline (PBS) solution containing 5% glycerol. The second series involved 0.01 M PBS solution containing 10% glycerol. Finally, a 0.01 M PBS solution containing 15% glycerol was used to wash the specimens. The samples were subjected to a decalcification procedure using an EDTA g solution (14.5 g EDTA, 1.25 g NaOH, and 15 mL glycerol) dissolved in distilled water with a pH of 7.3. The solution was subsequently prepared to a final volume of 100 mL and stored at a temperature of 4 °C for a duration of 10–14 days, following the methodology outlined by Mori et al. [25]. The EDTA g solution was replenished at regular intervals of 5 days. According to the established protocol, it is expected that complete decalcification of rat femur would typically occur within 10 days. Following decalcification, the bone was subjected to longitudinal sectioning and sliced into thin sections. The subsequent staining of these sections involved the utilization of hematoxylin, followed by eosin staining. Various specimens were observed using a microscope by Motic.

#### 2.4.5. Micro-Computed Tomography (µCT) Analysis of Bone

Micro-computed tomography (µ-CT) scans were conducted utilizing an ex vivo micro-CT scanner (μCT 40, SCANCO Medical AG, Brüttisellen, Switzerland). The scans were performed using a source voltage of 70 kV, a power output of 8 W, and a current of 114 μA, while maintaining an integration time of 200 ms. Approximately 60 slices, each of 12-μm-thickness, were determined in high resolution at 12 μm voxel size with 1000 projections. The assessment of bone mineral density (BMD), bone volume to tissue volume ratio (BV/TV), trabecular thickness (Tb. Th), trabecular number (Tb. N.), and trabecular separation (Tb. Sp) was conducted using a pre-installed software.

### 2.5. In Vivo Pharmacokinetic Studies

#### Animal Dosing and Sample Collection

The comparative assessment of pharmacokinetic parameters of RIS suspension, marketed formulation, RIS-HA-TCS nanoparticle, and RIS-HA-TCS-mPEG nanoparticles via the oral route was performed on Wistar rats procured from an animal house, Jamia Hamdard, New Delhi. Albino Wistar rats were divided into five groups, each with three rats. Groups 1, 2, 3, 4 and 5 were administered with normal saline, RIS-suspension, marketed preparation, RIS-HA-TCS and RIS-HA-TCS-mPEG nanoparticles, respectively. These preparations were administered orally to rats using an 18-gauge oral-feeding needle. The rats were subjected to anesthesia using diethyl ether, following which blood samples of 0.5 mL were withdrawn from the tail vein at specific time intervals (0, 1, 2, 3, 4, 8, 12, and 24 h). The blood samples were stored in microcentrifuge tubes pre-coated with ethylenediaminetetraacetic acid (EDTA). The plasma samples underwent separation through centrifugation at a speed of 10,000 rpm for 15 min. The plasma samples obtained were subsequently subjected to storage at a temperature of −20 °C in anticipation of drug analysis employing the reverse-phase high-performance liquid chromatography (RP-HPLC) technique, utilizing a binary pump manufactured by Shimadzu, Japan. The separation of RIS from plasma was facilitated with a mobile phase composed of a buffer solution containing 11 mM sodium phosphate, 5 mM TBAB ion-pair reagent, and 1.5 mM EDTA-2Na. Additionally, a 70:30 volume ratio of acetonitrile-containing mixture was used. This mixture of buffer and acetonitrile now has a pH of 6.75. At a flow rate of 1.0 mL/min, the sample was injected into the HPLC apparatus. A 5 μm size C-18 column (250 mm length and 4.6 mm diameter) was used as a stationary phase. The plasma samples were deproteinized by adding 450 µL of a 10% (*w*/*v*) trichloroacetic acid (TCA) solution and centrifuging the mixture at room temperature (1500 rpm) for 15 min. After separating the resulting supernatant, 50 µL of a 1.25 M CaCl2 solution and 57 µL of a freshly prepared 30% (*w*/*v*) NaOH solution were added. The resultant mixture was centrifuged at 4500 rpm for 10 min, while being vortexed. The plasma samples were again mixed with 400 µL of 1 M hydrochloric acid and heated at 90 °C for 30 min. The goal of this process was to help break down any pyrophosphates that might be in the rat plasma. The mixture was given an extra volume of 90 µL of a 30% solution of sodium hydroxide and 25 µL of a 1.25 M solution of calcium chloride. The resulting mixture was then put through a centrifuge for 10 min at a speed of 4500 rpm. The plasma samples were dissolved in a solution containing 50 µL of 1 M hydrochloric acid (HCl) and 2 mL of deionized water. Subsequently, a volume of 45 µL of a 30% NaOH solution was introduced into the sample, followed by centrifugation at a speed of 4500 rpm for a duration of 10 min. The solid substance was dissolved in 80 µL of a solution containing 0.025 M of EDTA-2Na. Subsequently, 920 µL of a liquid phase was introduced, and the resulting mixture was vigorously mixed, filtered, and analyzed using (HPLC) [26].

The experimental setup involved setting the wavelength to a specific value of 263 nm, while maintaining a constant run time of 10 min. The pump’s flow rate was set to 1 mL/min, injecting a 20 μL sample volume. Pharmacokinetic parameters, such as the maximum or peak plasma drug concentration (C_max_) and the time taken to reach the peak drug concentration in plasma (T_max_), were determined from the curve depicting the relationship between plasma drug concentration and time. The trapezoidal rule was employed to compute the area under the curves, namely AUC_0–24_ and AUC_0–∞_. The determination of the elimination rate constant (K_el_) involved analyzing the slope of the log plasma concentration plotted against time. Subsequently, the elimination half-life (t_1/2_) was calculated using Equation (1).

(1) $$t_{1/2}=\frac{0.693}{K_{el}}$$

### 2.6. In Vivo Histopathological Study of Organs

Wistar rats, with an average weight ranging from 225 to 250 g, were divided into five groups, each consisting of three rats. The experimental setup involved allocating groups. The control group (Group 1) received a standard saline solution. In contrast, Group 2 received a plain drug suspension (3.05 mg/kg), and Group 3 subjects (3.05 mg/kg) were administered marketed preparation. Groups 4 and 5 (3.05 mg/kg) were subjected to the administration of RIS-HA-TCS and RIS-HA-TCS-mPEG nanoparticles, respectively. After administering the dose, animals were sacrificed. Subsequently, tissue samples from the small intestine, liver, and kidney were extracted at 2, 8, and 24 h post-administration. These samples were then thoroughly rinsed with ice-cold saline solution, and subsequently preserved in a 10% buffer-formalin solution [27]. Sections with a thickness of 5 µm were obtained from each tissue sample. The sections underwent staining with a hematoxylin-eosin solution to facilitate histopathological examination. Subsequently, the blocks were subjected to observation using a light microscope, specifically the Digital Microscope-Miotic, BA310 Series.

### 2.7. Shelf-Life Determination

The stability investigations of the optimized RIS-HA-TCS and RIS-HA-TCS-mPEG formulations followed the principles outlined in the International Council for Harmonisation of Technical Requirements for Pharmaceuticals for Human Use (ICH) Q1A guidelines. The RIS-HA-TCS samples, along with RIS-HA-TCS-mPEG and the drug suspension, were subjected to storage conditions of 25 ± 2 °C and 40 ± 2 °C for a duration of 3 months. This storage was carried out in a stability chamber (TH 90 S, Thermolab, Mumbai, India). Samples were collected at various times, precisely at 0, 30, 60, and 90 days, and analyzed for their particle size distribution, polydispersity index (PDI), entrapment efficiency, precipitation appearance, and re-dispersibility [28].

## 3. Results and Discussion

### 3.1. Preparation of RIS -HA-TCS-mPEG Nanoparticles

The entrapment value of the nanoparticles, which were synthesized by combining RIS with HA in various ratios, exhibited variations. The results obtained from the RIS-HA experiment demonstrated a significantly higher drug entrapment value of 93.97% ± 1.56, indicating the successful encapsulation of a substantial amount of the drug within the system. Additionally, the experiment yielded a satisfactory value of 74.80% ± 2.61, suggesting an efficient production process with a relatively high output of the desired product. The selection of the optimized RIS-HA-TCS nanoparticles was determined via mathematical optimization technique using Design Expert^®^ software (Version 13.0, Stat-Ease Inc., Minneapolis, MN, USA). The primary criterion for selection was to achieve the maximum value of %EE (percentage encapsulation efficiency), while simultaneously minimizing the particle size and polydispersity index (PDI). By evaluating and comparing various responses using a numerical desirability function, it was determined that the formulation consisting of 1.5 mg/mL of TPP, 15 mg/mL of Drug-HA and 30 mg/mL of TCS (0.1:1:2) met the requirements of an optimized formulation. The nanoparticles of RIS-HA-TCS were optimized, resulting in the attainment of specific characteristics. The optimized nanoparticles’ particle size was 252.1 ± 2.44 nm, indicating a relatively small size. The nanoparticles’ polydispersity index (PDI) was determined to be 0.11 ± 0.01, suggesting a narrow size distribution and homogeneity. Additionally, the optimized nanoparticles’ encapsulation efficiency (%EE) was found to be 85.4 ± 2.21%, indicating that a high percentage of the drug has been successfully incorporated within the nanoparticles. The data for the above-mentioned method, characterization, and optimization of all parameters have been published in our previous work [19].

The optimized formulation was found to be an opalescent solution for which the stability was increased using mPEG. The formulation without methoxy polyethylene glycol (mPEG) was denoted as RIS-HA-TCS, while the formulation incorporating mPEG was designated as RIS-HA-TCS-mPEG. Additionally, particle size of the RIS-HA-TCS formulation was much larger than that of the RIS-HA-TCS-mPEG formulation. PDI of RIS-HA-TCS-mPEG formulation was lower, indicating that the particles were mono-dispersed. The entrapment efficiency of RIS-HA-TCS-mPEG exhibited a significantly higher value when compared to RIS-HA-TCS.

### 3.2. Confocal Laser Scanning Microscopy

**Figure 1:**
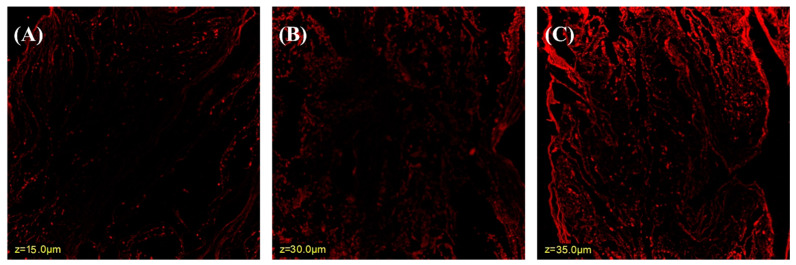
Confocal laser scanning microscopy of (**A**) control, (**B**) RIS-HA-TCS, (**C**) RIS-HA-TCS-mPEG.

The ability of the nanoparticles (NPs) to augment the absorption of Rhodamine B (Rh-B) from the intestinal barrier after oral administration was assessed using confocal laser scanning microscopy (CLSM). Figure 1 illustrates the confocal laser scanning microscopy (CLSM) images of three samples: pure Rhodamine B (Rh-B) solution, RIS-HA-TCS, and RIS-HA-TCS-mPEG. The CLSM findings revealed that the intestinal tissue treated with plain Rh-B solution exhibited fluorescence, limited to a depth of 15 µm (Figure 1A). In contrast, application of RIS-HA-TCS and RIS-HA-TCS-mPEG, both containing Rh-B dye, enhanced permeation and extended the fluorescence depth to 30 µm and 35 µm, respectively, as depicted in Figure 1B,C. The increased depth of permeation was observed in RIS-HA-TCS as compared to control due to the diminutive size of the particles, i.e., less than 160 nm, which eventually leads to larger surface area, and more permeation. However, better permeation was observed in the case of mPEG-coated nanoparticles as compared to uncoated nanoparticles despite their larger particle size [29,30]. The most probable reason behind it is the modulation of P-glycoprotein (Pgp) efflux transporter due to mPEG present in the coated nanoparticles. Henceforth, the outcomes obtained through confocal microscopy have demonstrated enhanced intestinal permeability of RIS when administered via RIS-HA-TCS-mPEG NPs.

### 3.3. In Vivo Pharmacodynamic Study

#### 3.3.1. Biochemical Estimation

After a treatment period of 4 weeks, blood samples were obtained from each rat in the respective groups and subjected to biochemical analysis.

##### Estimation of Calcium Level

**Figure 2:**
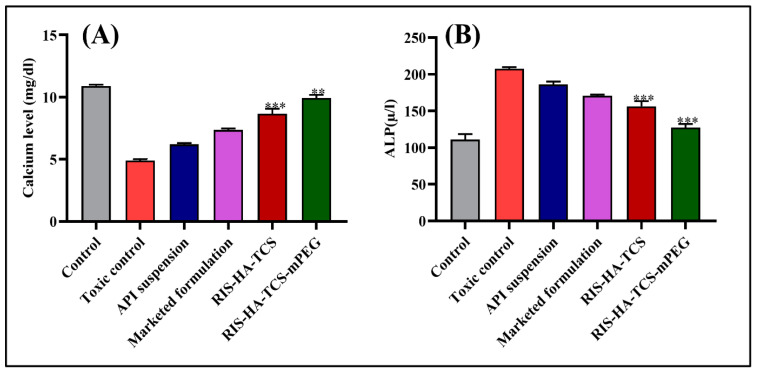
Biochemical estimation of (**A**) serum calcium level of different groups of animals, i.e., control, toxic control, API suspension, marketed suspension, RIS-HA-TCS, and RIS-HA-TCS-mPEG. (**B**) Serum ALP value of groups of animals, i.e., control, pathogenic, and treated with API suspension, marketed formulation RIS-HA-TCS and RIS-HA-TCS-mPEG using Graph pad prism. (** signifies *p* < 0.05, *** signifies *p* < 0.01).

The abundance of calcium in bone is crucial for maintaining bone mineral density (BMD). The serum calcium level was significantly lower in the toxic group (4.90 ± 0.10 mg/dL) compared to the normal control group (10.90 ± 0.10 mg/dL). Following treatment with RIS-HA-TCS-mPEG, the calcium level was found to be 9.93 ± 0.25 mg/dL, which was lower than the control group but higher than the RIS-HA-TCS group (8.67 ± 0.4 mg/dL), the marketed formulation group (7.37 ± 0.12 mg/dL), and the API suspension group (6.20 ± 0.11 mg/dL). As in the case of osteoporotic conditions, the bone becomes fragile, and the breakdown of bone is easy. Therefore, calcium flows through the blood and is excreted through urine, which significantly reduces calcium levels as glucocorticoids enhance urinary excretion and reduce intestinal absorption. The calcium concentration was significantly elevated in the experimental groups treated with RIS-HA-TCS-mPEG. This increase in calcium levels can be attributed to the preventive effects of RIS-HA-TCS-mPEG on bone resorption, which inhibits the breakdown of bone tissue. Furthermore, RIS-HA-TCS-mPEG has been shown to impede bone loss and promote bone mass accrual, ultimately contributing to the mitigation of bone fractures. These results are demonstrated in Figure 2A.

##### Alkaline Phosphatase Activity

Estimating serum alkaline phosphatase (ALP) is a biochemical indicator for assessing the process of bone turnover. It is a valuable marker of internal bone activity, and is used to track metabolic bone disease [31]. The serum ALP level significantly elevated in the toxic control group, i.e., DEXA-treated group 207.5 ± 2.1 µ/L (check units), as compared to the normal control group 110.9 ± 7.4 µ/L. The toxic group’s elevated ALP concentration revealed that osteoporosis had been induced. A considerable (*p* < 0.001) recovery was seen as the level of ALP reduced from toxic to control level after treatment; however, ALP level quickly dropped to 121.65.0 µ/L in the case of RIS-HA-TCS-mPEG-containing formulation, and to 156.4 6.9 µ/L in the case of RIS-HA-TCS. API suspension group (pure RIS treated group) and marketed formulation-treated group showed ALP levels of 186.2 ± 3.8 µ/L and 170.8 ± 1.4 µ/L, respectively. The API suspension-treated group and the marketed formulation-treated group showed a significant increase in the serum ALP level from normal, but below the toxic level due to the inhibitory effect of RIS. According to these results, RIS-HA-TCS-mPEG was able to reverse the effects of the induced bone loss by preventing bone resorption and promoting bone formation [32]. These results are demonstrated in Figure 2B.

#### 3.3.2. Microcomputed Tomography (µCT) Analysis

**Figure 3:**
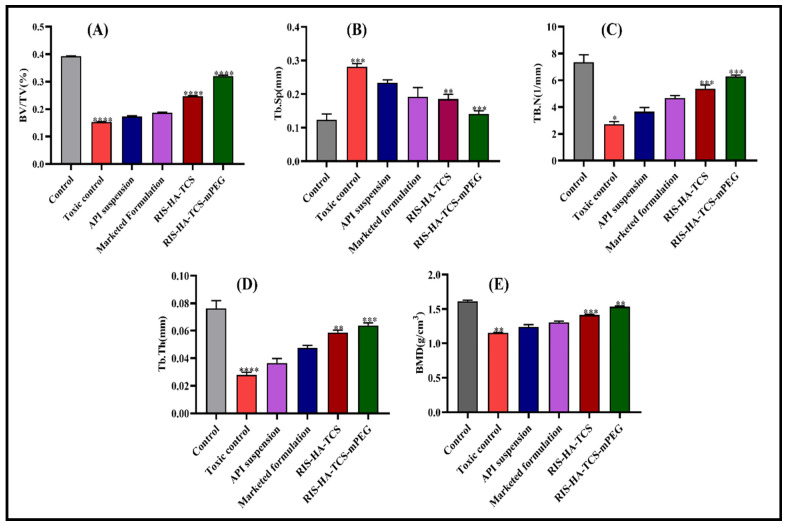
Comparative evaluation of microstructural trabecular bone parameters measured at distal femur region of various groups, (**A**) bone volume/trabecular volume, (**B**) Tb. N. tabular (1/mm), (**C**) trabecular spacing (mm), (**D**) thickness (mm), and (**E**) BMD bone mineral density (g/cm^3^). Data are presented as mean ± SD. (n = 3); Graph pad prism t-test (test vs. toxic control). (** = *p* < 0.05, *** = *p* < 0.01, **** = *p* < 0.001).

The bone architecture following treatment was evaluated using micro-CT, with a specific emphasis on morphometric parameters, including the bone volume to tissue volume ratio (BV/TV), trabecular number (Tb. N), trabecular spacing (Tb. Sp), trabecular thickness (Tb. Th), and bone mineral density (BMD). Based on the findings illustrated in Figure 3, notable alterations were detected in the trabecular bone structure of the distal femur. In the toxic control group, a significant reduction was observed in the bone volume fraction (BV/TV) values and trabecular number (Tb. N) compared to the control group. Furthermore, elevated levels of Tb. Sp was observed in the experimental group exposed to the toxic control (DEXA-treated group) compared to the control group under normal conditions. The administration of RIS-HA-TCS-mPEG exhibited significant enhancement in bone volume fraction (BV/TV), trabecular number (Tb. N), trabecular separation (Tb. Sp), trabecular thickness (Tb. Th), and bone mineral density (BMD) in comparison to RIS-HA-TCS, API suspension, and the marketed formulation group.

BV/TV, also known as fractional bone volume, is a widely employed parameter to evaluate osteoporosis-related bone deterioration. In Figure 3A, a significant reduction in the percentage of bone volume to total volume (BV/TV %) was observed in the toxic control group (0.153 ± 0.0015%) in comparison to the control group (0.398 ± 0.0012%) (*p* < 0.0001). This finding suggests the development of osteoporosis in the toxic control group. The prolonged administration of glucocorticoids, such as dexamethasone, has been shown to have detrimental effects on bone health. Glucocorticoids can reduce the replication of osteoblasts, which are responsible for bone formation, and impair their differentiation and maturation processes. Consequently, bone formation is diminished, leading to decreased bone density. Additionally, glucocorticoids have been found to increase the activity of osteoclasts, which are cells responsible for bone resorption. This further exacerbates the osteoporotic condition by promoting the breakdown of bone tissue [33]. As shown in Figure 3A, the group treated with RIS-HA-TCS and RIS-TCS-HA-mPEG showed BV/TV values of 0.248 ± 0.0027% and 0.320 ± 0.0016%, respectively, while the marketed group and API suspension group showed values of 0.187 ± 0.0015% and 0.173 ± 0.0026%, respectively. These findings indicated that the administration of both formulations resulted in a significant improvement in the medical condition. Nevertheless, it is essential to mention that the samples treated with RIS-TCS-HA-mPEG demonstrated a higher percentage of bone volume to total volume (BV/TV%) compared to the RIS-HA-TCS group. The observed increased value may be attributed to improved drug bioavailability from the RIS-TCS-HA-mPEG formulation. The improved bioavailability would eventually lead to a more effective delivery and distribution of the drug, resulting in a more significant therapeutic impact on bone health. Indeed, the higher BV/TV% value observed in the RIS-TCS-HA-mPEG-treated group suggested that this formulation might offer advantages in enhanced drug delivery and improved efficacy in treating osteoporotic conditions.

Trabecular number (Tb. N), expressed in units of mm^−1^, is a significant bone microarchitectural parameter commonly investigated in osteoporosis research. This study quantified the mean number of trabeculae intersected by a randomly placed line within the defined region of interest. The decrease in trabecular number precipitates alterations in bone microarchitecture, resulting in increased bone fragility. The concentration of Tb. N was found to be significantly reduced (*p* < 0.001) in the pathogenic control group (2.71 ± 0.21 mm^−1^) following the administration of dexamethasone, as compared to the control group (7.69 ± 0.53 mm^−1^). Tb. N was found to be significantly restored (*p* < 0.001) in the group treated with RIS-TCS-HA-mPEG (7.04 ± 0.20 mm^−1^), as well as in the group treated with RIS-TCS-HA (5.86 ± 0.10 mm^−1^) and the marketed formulation (4.85 ± 0.12 mm^−1^). The maximum restoration of bone was observed in the case of RIS-HA-TCS-mPEG NPs, which might be attributed to increased bioavailability as the required therapeutic concentration of the drug was maintained for a prolonged period through the oral route. Therefore, an enhanced pharmacodynamic action was seen (Figure 3B).

The relevant parameter for analysis was trabecular separation (Tb. Sp). The observed value of Tb. Sp, as depicted in Figure 3C, exhibited an increase in the case of the pathogenic control group (0.2816 ± 0.0092 mm) when compared to the control group (0.1235 ± 0.0175 mm). A significant decrease in Tb. Sp values were observed upon treatment with the RIS-TCS-HA-mPEG formulation (0.1412 ± 0.0091 mm), RIS-TCS-HA (0.18510 ± 0.014 mm), marketed-treated group (0.1915 ± 0.027 mm) and API suspension (0.2336 ± 0.0085 mm).

The trabecular thickness (Tb. Th) was found to be significantly higher in the control group (0.0762 ± 0.00561 mm) (Figure 3D) compared to the toxic control group (0.0277 ± 0.00194 mm). The trabecular thickness (Tb. Th) exhibited a significant enhancement in the RIS HA-TCS-mPEG treatment group (0.06383 ± 0.00188 mm) compared to other treatment groups, namely RIS-HA-TCS (0.05870 ± 0.00165 mm), API suspension (0.03651 ± 0.00322 mm), and marketed-treated groups (0.4757 ± 0.0018 mm).

As shown in Figure 3E, total bone mineral density (BMD) was found to be highest in the normal control (1.613 ± 0.0155 g/cm^3^) and lowest in the toxic control group (1.152 ± 0.0066 g/cm^3^). Dexamethasone-induced osteoporosis manifests in two distinct stages: an initial phase characterized by a swift decline in bone mineral density (BMD), likely attributable to increased bone resorption mediated by osteoclasts, followed by a subsequent, protracted phase defined by progressive BMD reduction due to compromised bone formation mediated by osteoblasts and osteocytes. The bone mineral density (BMD) in all groups treated with RIS was significantly higher than in those treated with pure API suspension. In comparison to the API suspension group (l.238 ± 0.0340 g/cm^3^), the RIS-HA-TCS-mPEG-treated group exhibited a higher BMD (1.533 ± 0.0116 g/cm^3^) than the RIS-HA-TCS group (1.413 ± 0.0105 g/cm^3^) and the marketed-treated group (1.306 ± 0.0192 g/cm^3^). Bisphosphonates have been widely acknowledged for their efficacy in treating osteoporosis, primarily attributed to their ability to enhance bone mineral density and mitigate the occurrence of bone fractures [34]. While bisphosphonates’ primary mechanism of action is the inhibition of osteoclastic bone resorption [35], an increasing amount of evidence suggests that bisphosphonates also engage with osteoblasts.

**Figure 4:**
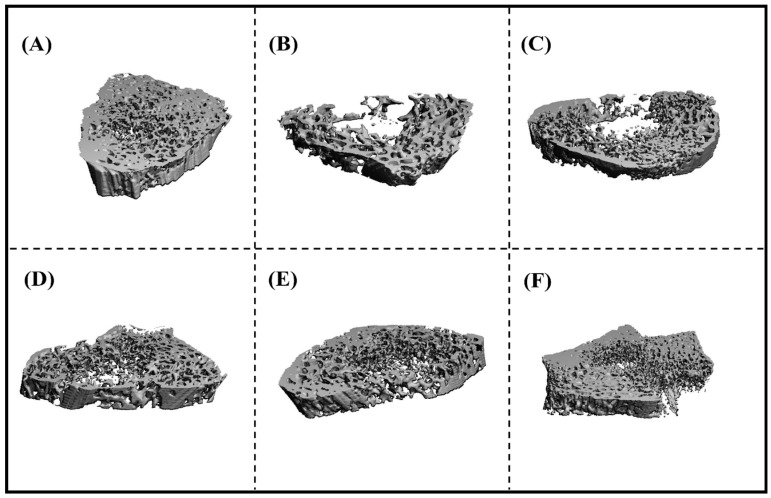
Image represents micro-CT analysis of different groups of treated animals. (**A**) Normal control, (**B**) toxic control, (**C**) API suspension, (**D**) marketed formulation, (**E**) RIS-HA-TCS AND, and (**F**) RIS-HA-TCS-mPEG.

The results from the micro-computed tomography (µ-CT study), as depicted in Figure 4, demonstrate the manifestation of trabecular microarchitecture degradation induced by dexamethasone. This deterioration was observed in the pathogenic control group (Figure 4B) compared to the control group (Figure 4A). The results of µ-CT demonstrated that the RIS-HA-TCS-mPEG-treated group had shown the best results compared to other treatment groups. The µ-CT analysis results indicated that the administration of RIS-HA-TCS-mPEG exhibited a statistically significant superiority compared to RIS-HA-TCS. It facilitated processes such as osteogenesis, the augmentation of trabecular bone density, and the reinforcement of trabecular bone connectivity, among other phenomena. The results mentioned below demonstrate that the utilization of RIS-HA-TCS-mPEG in oral drug delivery exhibits superior efficacy in the restoration of bone architecture and amelioration of the diseased state, in comparison to RIS-HA-TCS, the marketed available drug formulation, and the API suspension.

#### 3.3.3. Histology of Bone

**Figure 5:**
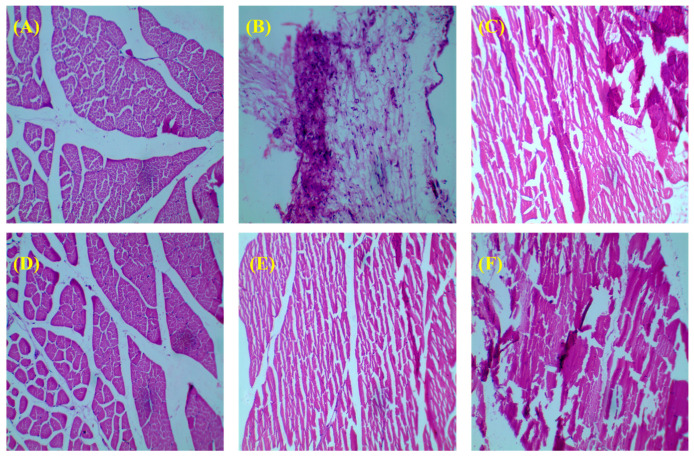
Histopathology of the femur bone. (**A**) Normal control, (**B**) toxic control, (**C**) API suspension, (**D**) marketed formulation, (**E**) RIS-HA-TCS nanoparticles, and (**F**) RIS-HA-TCS-mPEG (Olympus, 40× magnification).

The prolonged administration of bisphosphonates has been associated with modifications in the bone remodeling process [36]. The histological analysis of the femur specimens obtained from each experimental group of rats was conducted, and the resulting outcomes are presented in Figure 5. The control group exhibited the presence of typical dense micro-architecture within the diaphysis, along with compact trabeculae (Figure 5A). The femurs of the pathogenic control group rats showed significant trabecular thinning, absence of the growth plate, and expansion of intertrabecular gaps, which can be attributed to the loss of interaction between the bone structures. (Figure 5B). As compared to the toxic group, the histology of bone treated with RIS-HA-TCS-mPEG showed a marked restoration of the architecture of trabecular bone along with a well-connected bone matrix and narrow inter-trabecular spaces (Figure 5F) compared to RIS-HA-TCS (Figure 5E). Some positive changes (minor restoration) in the bone architecture of the femur were also observed in the case of marketed formulation-treated rats (Figure 5D) and API suspension-treated groups (Figure 5E).

#### 3.3.4. Histopathology of Other Organs

**Figure 6:**
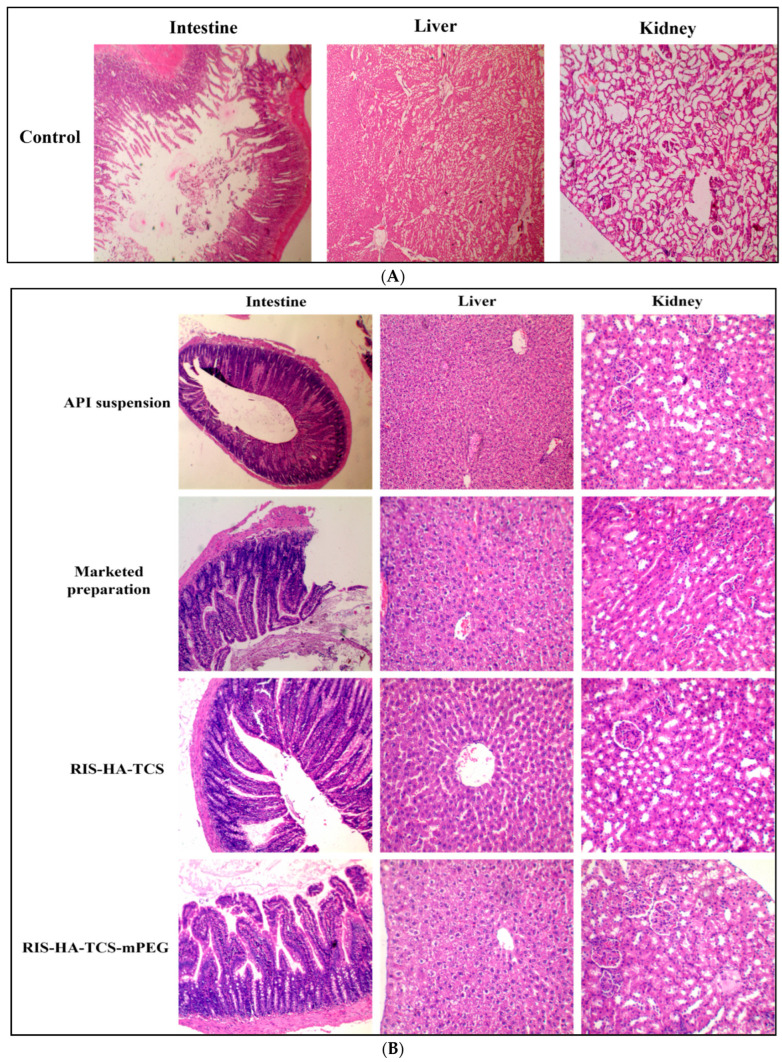

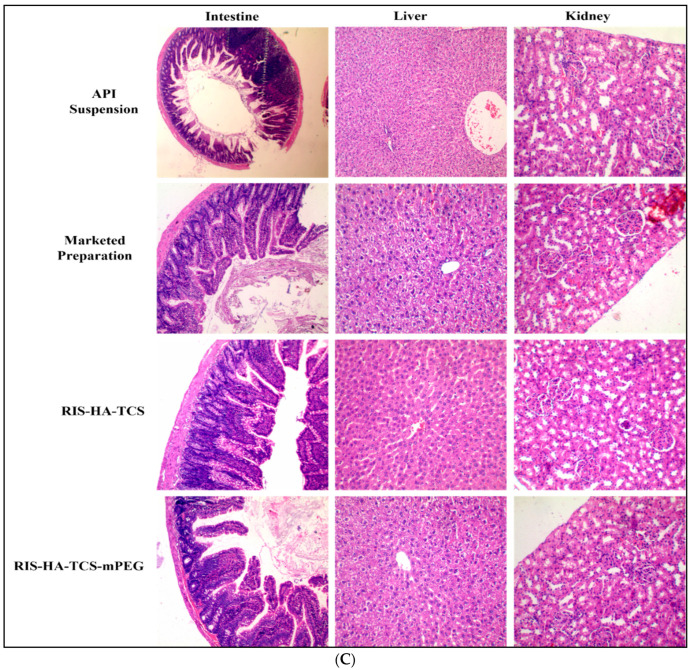

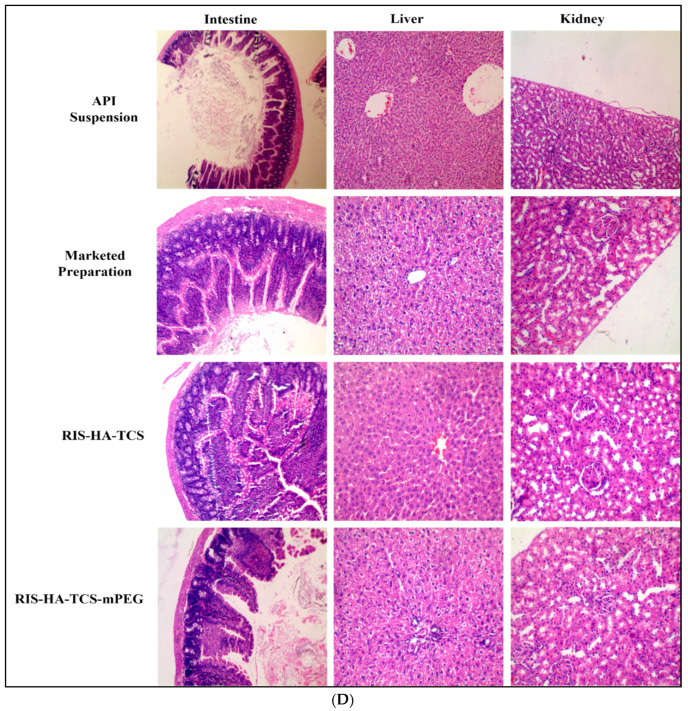
(**A**) Photomicrographs showing histology of normal saline-treated Wistar rats’ intestine, liver, and kidney (Olympus, 40× magnification). (**B**) Photomicrographs showing histopathological sections of the intestine, liver and kidney of Wistar rats after 2 h of oral administration of API suspension, marketed formulation, RIS-HA-TCS and RIS-HA-TCS-mPEG (Olympus, 40× magnification). (**C**) Photomicrographs showing histopathological sections of the intestine, kidney, and liver of Wistar rats after 8 h of oral administration of API suspension, marketed formulation, RIS-HA-TCS and RIS-HA-TCS-mPEG (Olympus, 40× magnification). (**D**) Photomicrographs showing histopathological sections of the intestine, kidney, and liver of Wistar rats after 24 h of oral administration of API suspension, marketed formulation, RIS-HA-TCS and RIS-HA-TCS-mPEG (Olympus, 40× magnification).

The histological examination of the intestinal, renal, and hepatic tissues revealed unremarkable epithelial linings devoid of discernible abnormalities. Lymph nodes were observed within the intestinal tissue, as depicted in Figure 6A.

After 2 h of treatment, liver segments obtained from the normal control group exhibited typical hepatic characteristics when examined histologically. These features were identified as numerous hepatocytes displaying round euchromatic nucleoli and a well-preserved sinusoidal lining (Figure 6A). As compared to the control group, liver tissues treated with API exhibited reduced cellular abnormalities, such as necrotic regions, vascular obstruction, and minor cellular degeneration. The renal and intestinal tissues exhibited negligible aberrations. The experimental group subjected to the RIS-HA-TCS treatment showed mild cellular damage, notable vascular congestion, and discernible cellular degradation within the hepatic tissues. The histological examination of the kidney and gut revealed no abnormalities in the tissue structure. From Figure 6B, it can be observed that the group treated with RIS-HA-TCS-mPEG did not exhibit any discernible abnormalities in the kidney, liver, and intestine tissues when compared to the other treatment groups. The group treated with the marketed formulation exhibited a notable lymph node enlargement within the intestinal segment, accompanied by the shedding of epithelial cells. Evidence of hemorrhage was observed within the hepatic tissue, and the renal tissue showed tubular dilation, glomerular shrinkage, and loss.

After an 8 h exposure to API suspension, notable sloughing and shrinkage were observed within the epithelial lining of the intestinal tissue. The tubular epithelial lining within the kidney tissue exhibited indications of debilitation and expansion, while the liver tissue demonstrated only slight cytoplasmic vacuolation and non-protein-related changes in hepatocytes. (Figure 6C). In contrast, the group treated with RIS-HA-TCS-mPEG exhibited no discernible abnormalities in the epithelial linings of the intestine. However, a minimal degree of tubule dilation was observed in the kidney tissues. Furthermore, the absence of necrotic lesions was noted in the hepatic tissue. In the experimental group subjected to the RIS-HA-TCS treatment, the examination of intestinal tissues revealed only minimal abnormalities, while the kidney tissue analysis indicated tubular dilation and weakening. The liver exhibited infiltration of peribiliary inflammatory cells, while the hepatic cells within the liver displayed a typical arrangement, as depicted in Figure 6C. The experimental group receiving the marketed formulation exhibited no significant alteration compared to the control group receiving the marketed product.

Following a twenty-four hours of treatment, it was observed that the groups subjected to drug suspension, marketed formulation, RIS-HA-TCS, and RIS-HA-TCS-mPEG nanoparticles did not exhibit any notable alterations in the histological characteristics of the intestine. The experimental results indicate that RIS-HA-TCS-mPEG lacked vacuolation in hepatocytes compared to RIS-HA-TCS, API suspension, and the marketed formulation (Figure 6D). No discernible abnormalities were observed in the renal tissue following the administered treatments. The results indicate that the prepared nanoparticles (NPs) exhibited no signs of liver cell necrosis. Evidence of epithelial cell thinning was observed in the group treated with the marketed formulation and the group treated with the active pharmaceutical ingredient (API) suspension. However, this phenomenon was not observed in the group treated with RIS-HA-TCS-mPEG, indicating that the designed nanoparticles are a safe carrier for facilitating the complete absorption of the drug through the oral route.

### 3.4. In Vivo Pharmacokinetic Study

**Figure 7:**
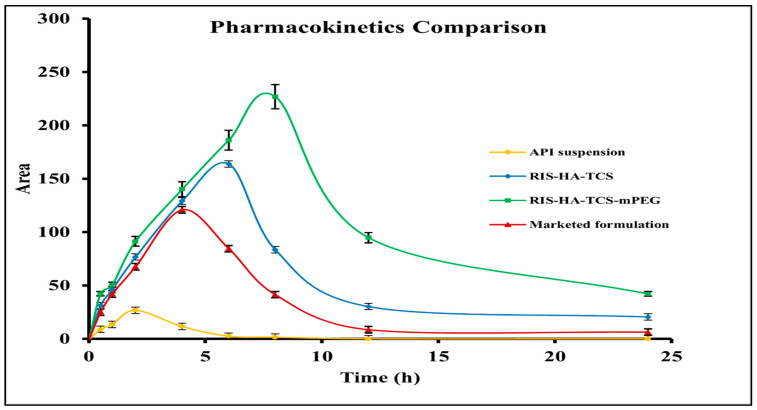
Comparison profile of drug suspension: marketed formulation, RIS-HA-TCS and RIS-HA-TCS-mPEG in pharmacokinetic study.

Albino Wistar rats subjected to treatment with the optimized formulation of RIS-HA-TCS-mPEG exhibited notably elevated plasma concentrations of RIS compared to rats treated with RIS-HA-TCS, marketed formulation, and RIS suspension (Figure 7). The observed differences in plasma concentrations were found to be statistically significant (*p* < 0.05) at all-time points that were assessed. The maximum plasma concentration (C_max_) of optimized RIS-loaded nanoparticles with PEGylation (RIS-HA-TCS-mPEG) was determined to be 226.84 µg/mL. This value was found to be significantly greater than the C_max_ values observed for the marketed formulation (127.38 µg/mL), RIS-HA-TCS (163.89 µg/mL), and API suspension (31.99 µg/mL). AUC_0→24_ of RIS-HA-TCS-mPEG was found to be 2299.53µg/mL*h, which was highly significant compared to RIS-HA-TCS’s AUC_0→24_ (1366.08 µg/mL*h), marketed preparation’s AUC_0→24_ (893.52 µg/mL*h) and RIS suspension’s AUC_0→24_ (266.67µg/mL*h). Small particle size, avoidance of first-pass metabolism, and increased RIS entrapment in the formulation contributed to the RIS-HA-TCS-mPEG nanoparticles’ much higher C_max_ and AUC_0→24_ due to the better absorption from the GIT and retarded progress toward the colon. There was a shift in t_max_ observed in the treated groups (RIS-HA-TCS-mPEG > RIS-HA-TCS > marketed preparation > and RIS suspension) because the PEGylated nanoparticles of chitosan took a relatively high time to get distributed in the plasma after oral administration than the chitosan nanoparticles and the marketed tablet. The low T_max_ for the treated groups (the drug suspension and marketed formulation) is also due to poor permeability of these formulations across the intestinal membrane [37]. The higher C_max_ observed for chitosan nanoparticles and the PEGylated nanoparticles is due to the prolonged contact of these formulations with the intestinal tissue and the spherical particle size in the nanometric range offering good permeation due to bioadhesion [38]. The higher t_1/2_ for the PEGylated nanoparticles also indicates a slow and gradual elimination from the blood stream with a considerably longer half-life (7.57 h). The increase in the MRT of PEGylated formulation is due to the use of stealth polymer which offers longer circulation in blood [39]. In comparison to drug suspension, relative bioavailability was found to increase in the order of 3.3, 5.1 and 8.6 fold for the marketed formulation, RIS-HA-TCS, and RIS-HA-TCS-mPEG, respectively [40,41]. The other pharmacokinetic parameters calculated are presented in Table 2.

### 3.5. Stability Studies

The optimized RIS-HA-TCS and RIS-HA-TCS-mPEG nanoparticles were subjected to a comprehensive evaluation encompassing several parameters. These parameters included the determination of precipitation occurrence, re-dispersibility, particle size (nm), polydispersity index (PDI) and entrapment efficiency (%EE). These evaluations were conducted at predetermined intervals to monitor any changes or trends. It was found that RIS-HA-TCS-mPEG was more stable than RIS-HA-TCS at 25 °C. RIS-HA-TCS-mPEG exhibited a particle size of (269.03 ± 0.9 nm), PDI of (0.160 ± 0.007), and entrapment efficiency o (83.93 ± 1.8%) after 90 days at 25 °C. However, there was a marginal increase in particle size over a storage period of 90 days. The experimental samples RIS-HA-TCS and RIS-HA-TCS-mPEG did not exhibit precipitation formation, and demonstrated favorable re-dispersibility even after a prolonged study period of 90 days. The findings derived from the stability studies are presented in Table 3.

## 4. Conclusions

The investigation into novel formulation techniques aiming to enhance the oral bioavailability of bisphosphonates and mitigate their gastric adverse effects has been presented. In this context, PEGylated risedronate hydroxyapatite-thiolated chitosan nanoparticles (RIS-HA-TCS-mPEG) were prepared using authentic sources of supply, and evaluated for their efficacy in osteoporosis treatment. Through a comprehensive in vivo evaluation in a rat model of osteoporosis, these nanoparticles demonstrated significant improvements in bone health compared to other formulations, including marketed products and the pure drug suspension. Moreover, the alterations in biomarker levels indicative of bone formation/resorption, and micro-CT analysis revealed the efficacy of RIS-HA-TCS-mPEG in promoting bone regeneration. The depth permeation through the intestinal wall for RIS-HA-TCS and RIS-HA-TCS-mPEG show 3-fold and 3.5-fold increase compared to the pure Rh-B dye suspension, respectively. Furthermore, histological assessments revealed a notable restoration of trabecular bone architecture, emphasizing the potential of this formulation to mitigate the adverse effects associated with oral RIS administration. Importantly, stability studies also confirmed the robustness of RIS-HA-TCS-mPEG, reinforcing its suitability for sustained drug delivery. In essence, the outcomes of this study underscore the significant therapeutic promise of mPEG-coated hydroxyapatite-based thiolated chitosan nanoparticles as a means to enhance the effectiveness of RIS therapy, while minimizing its side effects in the treatment of osteoporosis. This innovative formulation is a valuable contribution to the field of bone health, and holds great potential for improving the quality of life for individuals suffering from this debilitating condition. Further research and clinical investigations are warranted to validate and refine the application of these nanoparticles in osteoporosis management.

## Figures and Tables

**Table 1:** Animal grouping for the pharmacodynamic studies.

S.No.	Name of Groups	Dose	Route
A.	Control (normal saline)	-	Oral
B.	DEXA-induced Osteoporotic Model (dexamethasone)	4 mg/2 mL subcutaneously for 4 weeks, once per week	Subcutaneous(s.c.)
C.	DEXA-induced Osteoporotic Model + API suspension	3.05 mg/kg	Oral
D.	DEXA-induced Osteoporotic Model + Marketed preparation (Risofos, 35 mg/week)	3.05 mg/kg	Oral
E.	DEXA-induced Osteoporotic Model + RIS-HA-TCS	3.05 mg/kg	Oral
F.	DEXA-induced Osteoporotic Model + RIS-HA-TCS-mPEG	3.05 mg/kg	Oral

**Table 2:** Pharmacokinetic parameters calculated for the different treatment groups.

Parameters	Drug Suspension	Marketed Preparation	RIS-HA-TCS	RIS-HA-TCS-mPEG
C_max_ (µg/mL)	31.99	127.38	163.89	226.84
T_half_ (h)	2.80	4.61	6.74	7.57
T_max_ (h)	2	4	6	8
AUC_0–24_ (µg/mL*h)	266.67	893.52	1366.08	2299.53
AUMC_0–24_ (µg/mL*h^2^)	1644.59	6393.20	17,820.64	37,985.44
K_el_ (h^−1^)	0.24	0.15	0.10	0.09
MRT	6.1	7.16	13.05	16.51
F_rel_	-	3.3	5.1	8.6

**Table 3:** Stability data of the RIS-HA-TCS and RIS-HA-TCS-mPEG formulation at 25 ± 2 °C and 40 ± 2 °C for 90 days (n = 3).

Tem	Time (Days)	RIS-HA-TCS					RIS-HA-TCS-mPEG				
		Precipitate Formation	Re-Dispersibility	Particle Size± SD nm	PDI ± SD	% EE ± SD	Precipitate Formation	Re-Dispersibility	Particle Size ± SD nm	PDI ± SD	% EE ± SD
25 °C	0	NO	YES	252.10 ± 2.4	0.239 ± 0.015	85.03 ± 2.2	NO	YES	264.8 ± 1.9	0.120 ± 0.007	91.10 ± 1.3
	30	NO	YES	258.13 ± 1.19	0.242 ± 0.010	80.7 ± 1.8	NO	YES	266.0 ± 0.8	0.131 ± 0.002	89.60 ± 1.6
	60	NO	YES	263.4 ± 2.46	0.253 ± 0.010	73.5 ± 2.9	NO	YES	268.83 ± 0.7	0.146 ± 0.011	85.03 ± 2.2
	90	NO	YES	280.7 ± 2.7	0.268 ± 0.007	64.9 ± 2.6	NO	YES	269.03 ± 0.9	0.160 ± 0.007	83.93 ± 1.8
40 °C	0	NO	YES	252.10 ± 2.4	0.239 ± 0.015	85.03 ± 2.2	NO	YES	264.8 ± 1.9	0.120 ± 0.007	91.10 ± 1.3
	30	NO	YES	265.4 ± 1.91	0.267 ± 0.002	74.2 ± 1.7	NO	YES	269.5 ± 0.611	0.147 ± 0.011	89.10 ± 3.7
	60	NO	YES	217 ± 1.93	0.273 ± 0.003	65.1 ± 2.8	NO	YES	272.4 ± 0.88	0.183 ± 0.005	86.3 ± 0.6
	90	NO	YES	306.3 ± 2.67	0.285 ± 0.004	59.2 ± 1.4	NO	YES	280.3 ± 1.09	0.204 ± 0.021	79.2 ± 0.8

## Data Availability

The data presented in this study are available in this article.
